# Humeral and Glenoid Version in Reverse Total Shoulder Arthroplasty: A Systematic Review

**DOI:** 10.3390/jcm11247416

**Published:** 2022-12-14

**Authors:** Alessandra Berton, Umile Giuseppe Longo, Lawrence V. Gulotta, Sergio De Salvatore, Ilaria Piergentili, Giovanni Calabrese, Federica Roberti, Russell F. Warren, Vincenzo Denaro

**Affiliations:** 1 Research Unit of Orthopaedic and Trauma Surgery, Fondazione Policlinico Universitario Campus BioMedico, Via Alvaro del Portillo, 200-00128 Roma, Italy; 2 Research Unit of Orthopaedic and Trauma Surgery, Department of Medicine and Surgery, Università Campus Bio-Medico di Roma, Via Alvaro del Portillo, 21-00128 Roma, Italy; 3 Shoulder and Elbow Division of the Sports Medicine Institute, Hospital for Special Surgery, 535 E 70th Street, New York, NY 10021, USA

**Keywords:** reverse shoulder arthroplasty, reverse prosthesis, humeral version, glenoid version, range of motion, stability, muscle forces

## Abstract

There is increasing interest in reverse total shoulder arthroplasty (RTSA) as a reliable treatment for arthritic, rotator cuff deficient shoulders. Humeral and glenoid version are controversial parameters that can influence internal and external rotation, muscular forces, and implant stability as outcomes of RTSA. The aim of this study was to obtain an overview of the current knowledge on the effect of both humeral component version and glenoid component version and give recommendations on their most optimal degree for RTSA. A comprehensive quantitative review of the published literature on the effect of humeral version and glenoid version in RTSA was performed, to identify its influence on the range of movement, muscle forces, and intrinsic stability of the reverse prosthesis. Eleven studies were included: nine were biomechanical studies, one was a clinical-radiographic study, and one was an implant design consideration. Anterior stability can be improved by implanting the humeral component in neutral or with some anteversion. Glenoid component retroversion has been proven to reduce the likelihood of subluxation, while increasing ER and ROM at the same time. The study was conducted considering 5° anteversion; neutral; and 5°, 10°, and 20° retroversion of the glenoid component. Although a final opinion has not been yet expressed on the matter, the general consensus tends to agree on restoring 0° to 20° of retroversion of the humeral and glenoid component to yield the best outcomes.

## 1. Introduction

Reverse total shoulder arthroplasty (RTSA) has recently become a commonly accredited method for treating patients with shoulder pathologies, such as cuff tear arthropathy and degenerative diseases, reaching an incidence of 19.3 cases per 100,000 patients [1,2]. It has been shown how this technique is able to reliably improve pain and restore shoulder functionality, especially during elevation [3,4,5,6]. Moreover, its utility has been proven excellent in widely spread conditions as acute proximal humeral fractures, inflammatory arthropathy, and even in younger subjects [7,8].

Taking into account demographic aging and the continuous increment of the use of RTSA, it appears compelling to find new methods in order to ameliorate the outcomes.

Implant design and surgical technique can have a direct impact on outcomes, along with glenoid stem version and humeral stem version. In fact, the latter are parameters which can directly influence internal implant stability, muscular forces, and range of motion (ROM).

The practice bears high complication rates, such as scapular notching, which is notedly seen in 50–96% of patients [4,9,10,11,12,13,14], and prosthesis instability [4,15]. To contribute to the overall inaccuracy of the data reported regarding RTSA-related complications, variables such as the surgeon’s inexperience, patient-specific preoperative planning, and post-traumatic arthritis [16] were found to alter the perception of the outcomes of this technique. A decisive consensus has not yet been reached on the optimal degree of humeral version and glenoid version; although general parameters are beginning to be defined. Historically, technique guides suggested that the humeral stem be placed in 30° of retroversion, as is common for anatomic total shoulder replacements. However, recent literature suggests that less retroversion may be optimal to maximize rotation and improve stability [17,18,19,20]. As of today, few studies focus on the role of humeral version and glenoid version in RTSA. The aim of this study is to produce a review of the current literature about the implications of humeral version and glenoid version, due to the current paucity of reviews on the subject. Another purpose of the paper is to give recommendations on the most optimal degree of humeral component version and glenoid component version for RTSA.

## 2. Materials and Methods

### 2.1. Study Selection

The research question was formulated using a PICOS-approach: patient (P); intervention (I); comparison (C); outcome (O); and study design (S). The aim of this systematic review is to obtain an overview of the current knowledge on the effect of both humeral component version and glenoid component version and give recommendations on their most optimal degree for patients (P) who underwent RTSA (I). The comparator (C) was not assessed. The outcomes (O) assessed were impingement and ROM, muscle forces, and stability. The following study designs (S) were included: non-randomized controlled trials (NRCT), prospective (PS), retrospective (RS), comparative (CO), cohort (C), case-series (CS), systematic review (SR) basic science (BS), and clinical-radiographical (CR).

### 2.2. Inclusion Criteria

The criteria for the studies to be included were: biomechanical and clinical studies investigating reverse shoulder prosthesis and humeral and glenoid retroversion. All journals were considered, and all articles published in English were retrieved. Thus, the studies included focused on patients with irreparable rotator cuff tear, arthrosis, cuff tear glenohumeral arthropathy, glenoid B_2_ or C according to Walch classification, patients with abnormal bony architecture, and accompanying fracture.

### 2.3. Exclusion Criteria

Technical notes, instructional courses, letters to editors, or studies including procedures other than RTSA were excluded. Studies which were missing data were not included.

### 2.4. Search

Following the Preferred Reporting Items for Systematic Reviews and Meta-Analyses (PRISMA) guidelines [21], a comprehensive systematic review of the published literature on the effect of humeral version and glenoid version in RTSA was performed. Their influence was identified on range of movement (ROM), muscle forces, and intrinsic stability. Medline, EMBASE, Scopus, CINAHL, and CENTRAL bibliographic databases were accessed using the following keywords: humeral version, glenoid version, reverse total shoulder arthroplasty, retroversion reverse shoulder prosthesis. Articles cited by systematic reviews, as well as grey literature, were taken into consideration. The research yielded 570 results over the years 1985–2021.

The previously mentioned databases were accessed using the following search string: (((((“humerous”[All Fields] OR “humerus”[MeSH Terms] OR “humerus”[All Fields] OR “humeral”[All Fields]) AND (“version”[All Fields] OR “version s”[All Fields] OR “versions”[All Fields])) OR ((“glenoid”[All Fields] OR “glenoidal”[All Fields] OR “glenoids”[All Fields]) AND (“version”[All Fields] OR “version s”[All Fields] OR “versions”[All Fields]))) AND ((“reversal”[All Fields] OR “reversals”[All Fields] OR “reverse”[All Fields] OR “reversed”[All Fields] OR “reversely”[All Fields] OR “reverses”[All Fields] OR “reversibilities”[All Fields] OR “reversibility”[All Fields] OR “reversible”[All Fields] OR “reversing”[All Fields] OR “reversion”[All Fields] OR “reversions”[All Fields]) AND (“arthroplasty, replacement, shoulder”[MeSH Terms] OR (“arthroplasty”[All Fields] AND “replacement”[All Fields] AND “shoulder”[All Fields]) OR “shoulder replacement arthroplasty”[All Fields] OR (“total”[All Fields] AND “shoulder”[All Fields] AND “arthroplasty”[All Fields]) OR “total shoulder arthroplasty”[All Fields]))) OR “RTSA”[All Fields]) AND (2012:2021[pdat]). The research yielded 570 results over the years 1985–2021.

### 2.5. Data Collection Process

The titles and abstracts of all citations were reviewed by four investigators (A.B., P.F., F.R. and G.C.). The full manuscripts of articles satisfying the inclusion criteria and those of which the investigators were uncertain were downloaded. Reference lists of all full manuscripts and applicable review articles were reviewed to identify any further articles omitted from the initial search. The same investigators then reviewed all full manuscripts against the inclusion criteria, and any disagreement on eligibility was resolved by the authors. In order to design the PRISMA chart, standards mentioned by Moher et al. were adhered to when designing the PRISMA chart [22].

### 2.6. Study Risk of Bias Assessment

Given the designs of the included studies, the Risk of Bias in Non-Randomized Studies of Interventions (ROBINS-I) tool by Cochrane was used to verify the quality of each study [23]. The Risk of Bias (RoB_2_) tool for randomized trials [24] was not utilized since none were included in this review. The articles selected were independently rated by all reviewers (A.B., P.F., F.R., and G.C.).

## 3. Results

### 3.1. Study Selection

**Figure 1:**
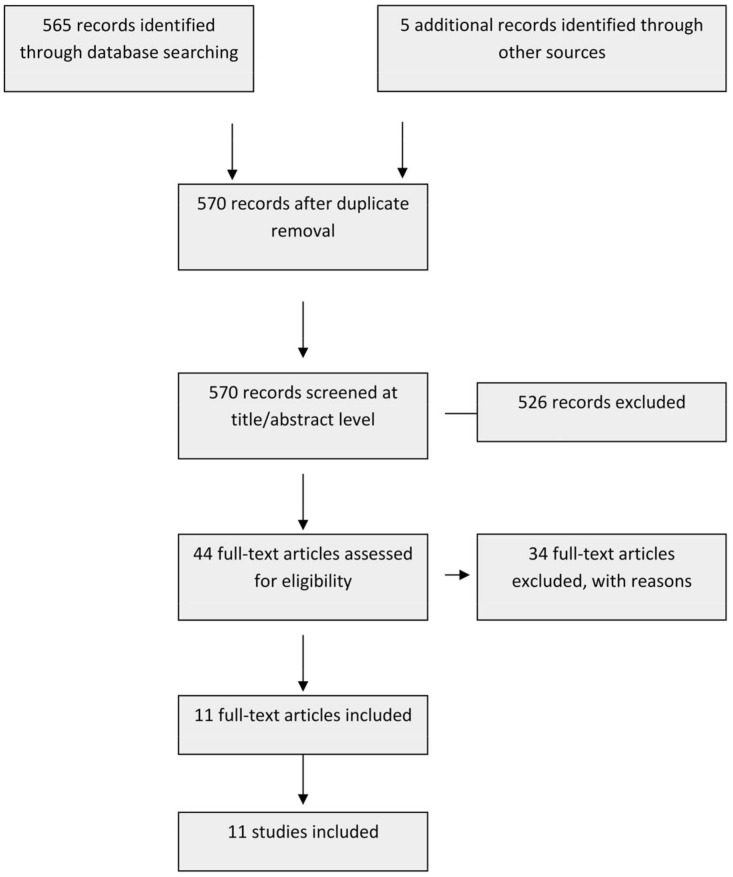
Flowchart of the screening selection.

The flowchart of the literature search is shown in Figure 1. In the literature search, 570 citations were identified and eleven studies met the selection criteria and were included in the review [17,18,19,20,25,26,27,28,29,30,31]. Many were excluded because they did not report data on the effects of neither humeral version nor glenoid version. Table 1 summarized the articles included in the review.

There were nine biomechanical studies [17,18,19,20,25,26,28,29,30]. Of these, five studies [17,20,25,26,30] assessed the effects of the humeral component version on range of movement and impingement of the humeral prosthesis against the scapula.

Conversely, there are two studies [18,28] focused on the effects of the glenoid component version on impingement and range of motion.

Two studies [19,29] were virtual biomechanical ones which assessed, respectively, how the different humeral version and glenoid version affect impingement and subluxation. Three studies [17,20,25] determined the effects of humeral component retroversion on muscle forces required to achieve specific motions. Two studies [18,25] analyzed the role of the humeral component version for intrinsic stability. Table 2 reports article conclusions on each topic.

There was only one clinical-radiographic study [27], as well as one implant design consideration [31].

### 3.2. Study Characteristics

The level of evidence (LOE) of the included studies was: one level II systematic review [31], one level II prospective comparative study [19], one level II retrospective cohort study [30], four level III basic science studies [17,18,25,26], one level III retrospective cohort study [29], one level IV case-series [28], and one level IV clinical-radiographic study [27].

Different prosthesis models were utilized in the studies: Delta III Total Reverse Shoulder Prosthesis (DePuy Inc, Warsaw, IN) [18,20] (DePuy International Ltd., Leeds, UK) [27]; Biomet Comprehensive Reverse Total Shoulder Replacement (Biomet Inc, Warsaw, IN, USA) [17,30]; and Aequalis Reversed Shoulder Prosthesis (Tornier, Edina, MN, USA) [25,26,29,30].

Regardless of the model of prosthesis used, there were no contradicting results in the studies.

The standard size of implant used among biomechanical studies was a 36 mm diameter glenosphere, with a margin of variation among different studies.

Three of the eleven studies implanted the prosthesis on shoulder specimens [17,25,26]. The humeral version degrees that have been tested were: 20° of anteversion, neutral version, and 10°, 20°, 30°, and 40° of retroversion. The glenoid version degrees that have been taken into consideration were 20°, 10°, 5° anteversion, neutral version, and 5°, 10°, and 20° retroversion.

The clinical-radiographic study [27] reported on 9 patients with 36 mm glenosphere and 18 patients with 42 mm glenosphere, with a mean glenohumeral component relationship of 34° (range, +6°–59°).

### 3.3. Quality of Evidence

**Figure 2:**
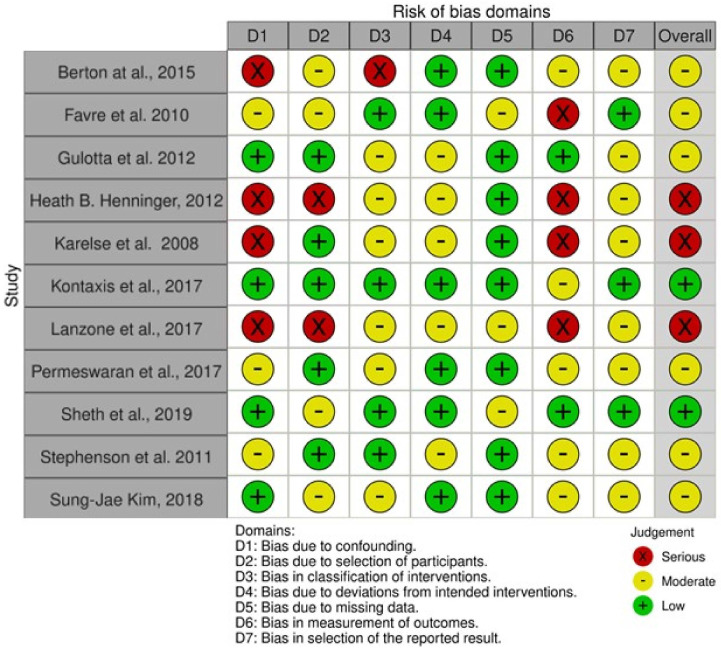
Risk of bias [17,18,19,20,25,26,27,28,29,30].

In order to assess the methodological quality of every article included in this review, the ROBINS-I tool was used for NRCTs [23]. Out of the total eleven NRCTs, two resulted as “low risk of bias” studies [19,31]; six resulted as “moderate risk of bias” studies [18,20,29,30]; and three resulted as “serious risk of bias” studies [25,27,28]. The assessments of the risk of bias of the NRCTs included in this study are reported in Figure 2.

### 3.4. Impingement and Range of Motion

Seven articles focused on the effect of humeral component version on impingement in reverse total shoulder arthroplasty [17,19,20,25,26,28,30].

Two of them used a custom-made biomechanical shoulder simulator [25,26], while four used a computer model [17,19,20,30].

Two studies [28,29] determined how variations in glenoid component version influence the arm abduction angle at which impingement occurs.

Stephenson et al. [26] tested all shoulders using a custom testing system, through rotational ROM at 0°, 30°, and 60° of glenohumeral abduction in the scapular plane. Each specimen was rotated through IR and ER until direct visualization of impingement was noted at four versions (20° of anteversion, neutral version, and 20°, and 40° of retroversion) at each abduction angle. They found that at 0° of abduction, IR and ER were both limited. Increasing humeral retroversion increased the amount of ER before impingement on the scapular border and decreased the amount of IR before impingement. There was no physiologic limitation to maximum IR at 30° of abduction. No impingement occurred at 60° abduction.

Henninger et al. [25] at first tested specimens in the native state and then with implanted reverse prostheses. They analyzed 0°, 10°, and 20° of humeral retroversion in abduction and ER ranges of motion. They found that an increased humeral retroversion resulted in an improved ER and consequent decreased IR. Retroversion of 10° exhibited the highest resting abduction angle (up to 40°). Increasing or decreasing retroversion by 10° decreased the resting abduction. Increasing or decreasing retroversion by 10° increased ER up to 10°.

Berton et al. [20] investigated the effect of humeral version on TM muscle moment arm, length, and impingement in RTSA during activities of daily living. A three-dimensional shoulder model was used to describe the surgical operation. They found that anteverted fixation maximized moment arm, but also increased inferior impingement (contact between the humeral cup and the inferior border of the scapula). In the same way, 40° humeral retroversion resulted in the largest TM muscle length, but also in the highest anterior impingement (contact between the humeral cup and the anterosuperior border of the glenoid). Moreover, 40° humeral retroversion displayed the largest ROM, while the 20° anteversion showed the smallest one. As a result, the humeral retroversion of 0° to 20° was the best compromise between adequate TM length and ROM, with the least impingement.

Gulotta et al. [17] used a three-dimensional computer model from computed tomography (CT) scans of the specimens and a corresponding 3D RTSA model created by laser scanning and virtually implanted into each specimen. The ROM in IR and ER was determined at 0°, 20°, 40°, and 60° of scaption, for each humeral retroversion (0°, 20°, 30°, and 40°), using a specific 4D software. They found that all cadavers showed 0 ROM at 0° scaption due to abutment of the humeral bearing to the inferior neck of the glenoid. At 20 and 40 of scaption, as retroversion was increased, the relative amount of ER increased as well, with a loss of IR. At 60° of scaption, the models were able to rotate internally and externally more than 90° without bony impingement.

Kim et al. [30] performed an analysis of impingement-free ROM of the glenohumeral joint after reverse total shoulder arthroplasty. They used 3D scapulohumeral models obtained from preoperative CT images and three types of implant designs for reconstruction. It was shown that increasing humeral retroversion (between 0° and 20°) is significantly related to increased external (ER) and decreased internal rotation (IR). Furthermore, increased humerus retroversion might be related to increased abduction.

Kontaxis et al. [19] determined how humeral version affects impingement during activities of daily living. The study included thirty virtual arthritic shoulders which were reconstructed from preoperative CT scans. The results drawn from the investigation confirm that 0° version led to the least amount of impingement, while a retroversion of 40° allowed the largest ROM. A maximized ROM does not necessarily reduce the occurrence of impingement; therefore, the authors suggest an average of 0° humeral version.

Permeswaran et al. [29] investigated how variations in glenoid version influence the arm abduction angle, possibly resulting in impingement. The study was performed on thirty-five computer models taking into consideration 5° anteversion; neutral; and 5°, 10°, and 20° retroversion of the glenoid component. The authors found that retroversion reduces the likelihood of subluxation, while increasing ER and ROM at the same time.

Lanzone et al. [28] tested twenty patients with cuff tear arthropathy and glenoid retroversion greater than 15 °, assessed according to the Walch classification. The mean preoperative retroversion of the glenoid was 24°, while the post-operative was 2°. This confirms the ongoing evidence that restoring glenoid version around 0° yields the most optimal outcomes.

Favre et al. [18] obtained similar conclusions.

### 3.5. Muscle Forces

Three studies [17,20,25] focused on retroversion influence on muscular forces to abduct the arm.

Berton et al. [20] investigated the ROM and TM muscle length, as well as six common activities of daily living. From the study it was evident that 40° humeral retroversion allowed for the maximum TM muscle length. Nonetheless, the same value of humeral retroversion led to the highest anterior impingement. As a result, the humeral retroversion of 0° to 20° was the best compromise between adequate TM length and ROM, with the least impingement.

Gulotta et al. [17] used a custom-made shoulder simulator that uses a physiologically based optimization algorithm to determine the individual muscle forces (subscapularis, teres minor, anterior deltoid, middle deltoid, posterior deltoid, latissimus dorsi, pectoralis major) required to achieve 30° and 60° of scaption for each retroversion (0°, 20°, 30°, and 40°). No significant differences were found among retroversions for any muscle, including the teres minor. The most force was provided by the middle deltoid followed by the anterior deltoid and the posterior deltoid. An almost negligible force was provided by the latissimus dorsi and the pectoralis major. The contribution of the subscapularis was roughly equivalent to that of the teres minor.

Similar results were collected by Henninger et al. [25]. They used a biomechanical shoulder simulator consisting of pneumatic cylinders that applied displacement to the deltoid insertion to abduct the arm in the scapular plane while load cells recorded force. Static loads were applied to the insertions of the rotator cuff muscles. A three-dimensional optically tracking diode arrays on the fixation pins allowed quantifying arm kinematics. Tests were performed both with straight and flexed elbow. They found that reverse arthroplasty cases required significantly less force to abduct than the native arm. The cumulative deltoid force was 30% lower than in native arms. However, it did not change when the version was altered.

### 3.6. Stability

Two studies [18,25] investigated the role of humeral retroversion in the stability of the reverse shoulder arthroplasty.

Favre et al. [18] carried out mechanical tests on the stability of the implant with different combinations of humeral version and glenoid version (20°, 10° anteversion, neutral, 10°, 20° retroversion) at 90° and 20° of humeral abduction. They applied a constant compressive load of 40 N from medial to lateral, and a dislocation force from posterior to anterior. They measured the resistance to dislocation by the stability ratio (peak force in the load-displacement curve divided by the axial compressive load). The stability ratio for corresponding configurations was higher in the 90° abducted position than in the resting position. A change of 10° in humeral component version affected the stability ratio more than an identical alteration in the glenoid component (respectively, 21% and 5% in 90° of abduction, and 27% and 15% in resting position). The standard implant configuration (neutral glenoid version and 20° humeral retroversion) yielded the second worst stability ratio of all tested configurations and could only be increased by anteverting the humeral component.

Henninger et al. [25] tested the force to dislocate the implant on a shoulder simulator with a 2% body weight load and provided by load cell that recorded laterally (with the arm at neutral ER) and anteriorly (with the arm at 90° ER) directed force. The force required for lateral dislocation was 60% less than anterior and was not affected by implant version.

### 3.7. Clinical Study

Only one study [27] analyzed the relationship between the position of the prosthetic components and rotation in the clinical setting. Twenty-seven patients, who received a Delta III Reversed Total Shoulder Prosthesis (36 mm glenosphere in 9 patients, 42 mm in 18 patients) for cuff tear arthropathy were studied (mean follow-up, 43 months) using active and passive range of motion, Constant–Murley score, standard radiographs, and computed tomography. A uniform spatial reference system was used to analyze the position of the prosthetic components radiologically in the transverse plane. The angle between the axes of the glenoid and humeral components was described as anterior divergence (humeral stem retroverted) or convergence (humeral stem anteverted). Internal–external rotation was not only clinically studied but also radiologically, measuring the difference between the axes of the humeral component in the prone (b) and supine (a) positions. They reported a significant correlation between increased anterior divergence and radiologically measured IR. Instead, there was no correlation between the anterior divergence and the degree of passive and active ER, nor any correlation with impingement, notching, and spur formation. The lack of active ER was not correlated with the fatty degeneration of the teres minor muscle. Moreover, there was no statistically relevant correlation between divergence and clinically measured IR. However, patients with a mean divergence of 34° had powerful IR that can probably be explained by the remaining functional internal rotators (major pectoralis muscle, latissimus dorsi, and teres major).

### 3.8. Implant Design Consideration

Among the literature included, one study [31] provided an overview of the different RTSA designs and outcomes related to both humeral and glenoid components. The study states that, traditionally, the optimal degree of humeral version is 0° to 30° of retroversion. According to Stephenson et al. [26], who conducted a cadaveric study, a 20° to 40° of humeral retroversion led to the most optimal outcome restoring functional ROM, with minimum impingement. Other studies [32,33], mentioned by Sheth et al. [31], confirmed that increased humeral retroversion results in a decreased IR and increased ER. Lastly, one study [31] reports the conclusions drawn by Rhee et al. [34], similarly to Kontaxis et al. [19], stating that 0° of humeral retroversion allowed patients to perform remarkably better in activities of daily living.

## 4. Discussion

The main finding of this study is that a humeral retroversion of 0° to 20° and a glenoid retroversion of 0° to 15° seem to yield the best outcomes in RTSA.

Although RTSA has been commonly accepted as a reliable treatment for arthritic, rotator cuff deficient shoulders, not as much evidence exists in the literature to direct implant design and surgical technique. Although a final opinion has not yet been expressed on the matter, the consensus tends to agree on restoring 0° to 20° of retroversion of the humeral and glenoid component to yield the best outcomes [19,20,28,29,31,35].

Several aspects of RTSA, such as glenosphere size and vertical position, humeral component version and glenoid component version, as well as inclination angle, influence a variety of factors, such as ROM, impingement, muscular strength, and stability; thus, reflecting on successful outcomes [17,18,19,20,25,26,29,30,31,36,37,38,39].

In this review, the literature focuses on humeral component version and glenoid component version in RTSA. Eleven articles were included in the review. These studies mainly focused on the contribution of humeral version to the amount of IR and ER achieved after RTSA [17,25,26]. They also investigated the effect of humeral version on muscular forces [17,20,25] and implant stability [18,25].

Gulotta et al. [17] found that impingement between the implant and the scapular neck is most pronounced with the arm at low levels of abduction and that this impingement becomes less important as the arm is elevated. Moreover, poor scapulothoracic mobility does not play a role in limiting shoulder abduction, but maintains overall functionality in RTSA patients [40]. Sheth at al. [31] confirmed that humeral retroversion of 20° to 40° restored the physiological functional arc of motion, with no impingement involved. Considering activity of daily living, IR is important with the arm at the side, such as reaching the back pocket, performing perineal hygiene, washing the back, and pushing off a chair to rise from the sitting position. Meanwhile, ER is important when the arm is elevated, such as putting the hand to the mouth in order eat, washing hair, and removing items from shelves that are above the head [19,41]. A study performed by Kontaxis et al. [19] on the effects of humeral version on impingement in daily living activities revealed how 0° version displayed the least amount of impingement, while 40° retroversion allowed for the largest ROM.

Muscle forces, when compared to impingement, play a far more relevant role in order to externally rotate the shoulder. For these reasons, Gulotta et al. [17] recommended placing the implant in 0° to 20° of retroversion to maximize IR with the arm at the side, and performing a concomitant latissimus dorsi transfer in patients with a deficient teres minor to obtain functional ER with the shoulder in abduction [42,43,44].

Stephenson et al. [26] had similar results, but slightly different recommendations. They advised to place the humeral component between 20° and 40° of retroversion to maximize impingement-free ROM and possibly subluxation or dislocation with ER. Concurrently, Permeswaran et al. [29] have shown how a neutral glenoid component version is associated with the least occurrence of subluxation. Moreover, the same study proved how rotating modern radially asymmetric humeral polyethylene liners in a posterior fashion can decrease the likelihood of subluxation (which may lead to dislocation) and increase ER and ROM.

Henninger et al. [25] compared abduction and ER ranges of motion between native arms and implanted arms. The authors showed that increasing humeral retroversion resulted in an improved external rotation (ER), with a diminished internal rotation (IR).

The same topic was recently further explored by Kim et al. [30]. The study has proven how increasing humerus retroversion (between 0° and 20°) significantly increases ER while, at the same time, decreasing IR. Furthermore, increased humerus retroversion might be related to increased abduction.

However, the studies carried out by Henninger et al. and Stephenson et al. [25,26] have some limitations. In fact, each of them only used one type and size of prosthesis. As a result, studies that investigate the same topic through varying sizes and implant designs [29,30] might yield more accurate outcomes.

These elements influence impingement due to humeral bearing, lateralization of the glenosphere, and neck-shaft angle of the humeral component. The Grammont-style prosthesis was used in all of the studies, but the designs differ from each other. This may explain some differences in the results. Moreover, no study considered impingement between the soft tissues and the scapula, which may be clinically relevant and must, therefore, be taken into consideration when investigating the topic.

Muscle forces across the shoulder joint during scaption have been analyzed for different degrees of humeral version. Henninger et al. [25] found that deltoid abduction force was reduced by 30% after reverse TSA. This decrease in abduction force occurred regardless of the humeral version or implant thickness, and it was the result of the medial/inferior shift of the humeral center of rotation. Gulotta et al. [17] confirmed their results and concluded that humeral retroversion does not provide mechanical advantage of the teres minor and posterior deltoid during elevation. Both studies used a shoulder simulator to reproduce muscle action forces. Despite the fact that such simulators are widely regarded as reliable and accurate, they bear some limitations which might reduce the ability to describe the in vivo conditions. For instance, muscle forces are active and dynamically changing, while these studies only measured muscle forces under static conditions.

Studies also focused on implant stability, as it represents an important key point for the high rate of dislocation observed in RTSA [4,29,45,46,47,48]. Intrinsic stability is essential because of the lack of muscular stabilizer [9,43]. Henninger et al. [25] confirmed that RTSA stability did not seem to be affected by version. Intraoperatively, Gavaskar et al. [49] demonstrated that CT navigation may be a relevant means for ensuring the initial stability of the glenoid component. Favre et al. [18] found that the humeral component version has a greater influence on intrinsic stability than the glenoid component version. Anterior stability can be improved by implanting the humeral component in neutral or with some anteversion. Those data are accompanied by favorable clinical outcomes [27].

However, the biomechanical studies mentioned [18,25] have some limitations. Only one design of reverse prosthesis was used and the influence of socket depth on intrinsic stability was not considered. Moreover, in vivo forces causing dislocation of the reverse prosthesis are yet to be investigated, as well as the stabilizing or destabilizing effect of muscles, ligaments, and capsule, which were impossible to model.

Only one study [27] reported information about the effect of the humeral version on clinical and radiographic outcomes of RTSA. Radiologically measured passive IR with the arm adducted is increased when divergence of the components is higher. However, there is no significant influence on the degree of passive and active external rotation. In order to achieve the optimal position of prosthetic components, a spatial reference system of axes in the sagittal or coronal plane of the body should be used.

The study conducted by Sheth et al. [31] states that, traditionally, the optimal degree of humeral version is 0° to 30° of retroversion. According to Stephenson et al. [26], who conducted a cadaveric study, a 20° to 40° of humeral retroversion led to the most optimal outcome restoring functional ROM, with minimum impingement. Other studies [32,33], mentioned by Sheth et al. [31], confirmed that increased humeral retroversion results in a decreased IR and increased ER. Lastly, one study [31] reports the conclusions drawn by Rhee et al. [34], similarly to Kontaxis et al. [19], stating that 0° of humeral retroversion allowed patients to perform remarkably better in activities of daily living.

### 4.1. Limitations

This systematic review presents some limitations, such as the relative lack of literature on the topic. Moreover, a considerable number of the studies published offer a broader, not as specific, perspective on this matter, since their focus is on the comparison between TSA and RTSA. Furthermore, the majority of the papers published on this topic are retrospective studies, which may not be fully successful in retrieving past data, such as patient information. Analogously, they may not include follow-up reports regarding clinical implications, such as how scapulothoracic joint influences shoulder mobility and how glenohumeral joint rotation elevation affects impingement. Lastly, few to no studies at the present focus on young individuals and are, therefore, influenced by concurrent iatrogenic conditions, which may ultimately affect the results of the study itself.

### 4.2. Strength

The relevance of this systematic review is to have elucidated the current knowledge regarding the most optimal degree of humeral component version and glenoid component version for RTSA, since these factors influence impingement-free range of movement, muscle forces, and implant stability.

Conversely, in recent years, a surge of published studies on the topic has been seen. This is the testimony of a growing interest that will inevitably lead to a better and more thorough understanding of the matter. Moreover, a general consensus regarding the humeral component version and glenoid component version has recently become clearer.

## 5. Conclusions

Humeral component version and glenoid component version seem to affect the outcomes of RTSA, together with the patient’s ability to perform daily activities. Substantial progress has lately been achieved regarding this matter. As of today, multiple variables exist on both the humeral and glenoid side that contribute to maximizing implant efficiency. Although the current literature has not yet reached an unequivocal consensus, the common knowledge seems to favor 0° to 20° of humeral retroversion and 0° to 15° of glenoid retroversion to obtain the best outcomes in RTSA.

Following the recent interest on the topic, future biomechanical and clinical studies can be expected to draw a clear-cut, definitive guideline on the degree of humeral component version and glenoid component version in this surgical technique.

## Figures and Tables

**Table 1:** Study characteristics.

Author, yr	Type of Study/LOE	Aim	Sample Size	Sample Anteversion/ Retroversion	Size and Type of Implant	Version Degrees	Abduction/Scaption Degrees	Conclusion
Berton et al., 2015 [20]	CS, IV	To investigate how the version of humeral fixation can affect the TM rotational moment arm and muscle length as well as impingement after RTSA	A3-dimensional shoulder model		Adapted version of the Newcastle Shoulder Model that resembles the geometry of a commercially available reverse shoulder prosthesis Delta III prosthesis (DePuy International, Leeds, UK)	Four humeral fixation versions were tested: +20°, 0°, −20°, and −40° (+anteverted; -retroverted)	Humeral abduction in scapular plane from 0° to 150°)	The 0 and 20 retroversion was the optimum compromise between sufficient TM length and moment arm with minimum impingement
Favre et al., 2010 [18]	BS Study, BcS	Effects of component positioning on intrinsic stability	150 trials		Delta III RTSA (DePuy Orthopaedics, Inc., Warsaw, IN, USA) size 36	Version of the glenosphere: from 20° retroversion to 20° anteversion in 10° steps. Version of the humeral component: neutral, 10°, 20° retroversion and anteversion	20° and 90° abduction	Effect of humeral component version was highly significant. Implanting the humeral component in neutral or some anteversion can improve anterior stability
Gulotta et al., 2012 [17]	BS Study, BcS	Effects of humeral component retroversion on the muscle forces across the shoulder joint during scaption			Biomet Comprehensive RTSA (Biomet Inc, Warsaw, IN, USA)	0°, 20°, 30°, and 40° of retroversion	30° and 60° of scaption	Increasing retroversion did not affect the muscle force
		Impingement-free IR and ER allowed for varying humeral retroversions			9 mm stem, cemented, neck-shaft angle 147°, 36 mm glenosphere			Placing the implant in 0° to 20° of retroversion maximizes IR with the arm at the side. Concomitant latissimus dorsi transfer is recommended for patients with a deficient teres minor
Henninger, 2012 [25]	BS Study	Effects of humeral version and deltoid tension on (1) ROM in glenohumeral abduction, degree of ER of a flexed arm		6 cadaveris shoulders (retroversion)	Aequalis Reverse Shoulder (Tornier, Edina, MN, USA) 6.5 mm humeral stem, 36 mm glenosphere	0°, 10°, 20° of retroversion. 9 combinations of humeral version and implant thickness	Resting abduction and 60° scapular abduction	RTSA reduced ER compared with intact shoulder. Deltoid tension and humeral version had little effect on ER
		(2) force required to abduct the arm					From resting abduction position to 65° of glenohumeral abduction in the scapular plane	Humeral version and implant thickness did not influence abduction forces
		(3) force required to dislocate the implant in a lateral or anterior direction					Resting abduction position in neutral and 90° ER	Version and deltoid tension did not significantly alter dislocation forces
Karelse et al., 2008 [27]	RSC Study, II	Relation between the positioning of the components in the transverse plane and its consequences on IR/ER of the shoulder	27 patients		Delta III prosthesis (DePuy International, Leeds, UK) 36 mm glenosphere in 9 patients, 42 mm glensphere in 18 patients	Mean glenohumeral component relationship: 34° with variation from +6° to 59°.	Arm adducted in the coronal plane	To achieve the optimal position of the prosthetic components it should be used a spatial reference system of axes in the sagittal or coronal plane of the body
Kontaxis et al., 2017 [19]	PS CO study, II	To determine how humeral version affects impingement in activities of daily living	30 virtual arthritic shoulders (Mean age: 71.6 years)	30 virtual arthritic shoulders (anteversion) 30 virtual arthritic shoulders (retroversion)	Comprehensive RTSA (Biomet, Inc., Warsaw, IN, USA) 28 mm diameter glenoid baseplate; 36 mm diameter standard glenosphere without offset; size 4 humeral “platform” long stem; 44 mm diameter standard onlay humeral tray; and a 44 to 36 mm diameter standard humeral bearingThe neck/shaft angle of the prosthesis and the humeral cut was at 135°	Each of the 30 arthritic shoulders were placed into 5 versions: −40°,−20°, 0°, +20°, and +40°	Largest impingement-free abduction (118° ± 19°) found in −40° version; smallest abduction (19° ± 13°) found in +40° version	Maximizing ROM in standardized tests may not reduce the risk of impingement. An average 0° of humeral version should be preferred
Lanzone et al., 2017 [28]	CS, IV	To evaluate the clinical and radiological results of RSA with glenoid plating in a series of patients affected by cuff tear glenohumeral arthropathy with glenoid retroversion >15°	20 patients (Mean age: 75 ± 5 (61–81))			Preoperative mean glenoid retroversion: 24° (range 15°–38°, SD 7) Postoperatove mean glenoid retroversion: 2° (range 0°–7°, SD 2)		Retroverted glenoid reconstruction with glenoid plates in RSA is an alternative method to address severe glenoid deficiency
Permeswaran et al., 2017 [29]	RSC study, III	To determine how changes in glenoid component version affect the arm abduction angle at which impingement occurs	35 different clinically representative models (computer modeling)	7 virtual shoulders (anteversion) 21 virtual shoulders (retroversion)	Aequalis Ascend Reverse Flex convertible shoulder system Computer models of the 36 mm Aequalis Ascend Reverse Flex implant system	5° anteversion; neutral; 5°, 10°, and 20° retroversion	35° of abduction during flexion to replicate arm swinging	Rotating modern radially asymmetric humeral polyethylene liners posteriorly can reduce the risk of subluxation leading to dislocation and increase ER range of motion
Sheth et al., 2019 [31]	SR, III	To provide a review of RTSA designs						Multiple implant-related variables exist on both the humeral and glenoid side that play a role in maximizing implant efficiency
Stephenson et al., 2011 [26]		Effects of humeral component version on impingement of the humeral prosthesis against the scapula	7 cadaveric shoulders		Aequalis Reverse Shoulder (Tornier, Edina, MN, USA) 6.5 mm stem, 36 mm metaphyseal component, a 36 ± 9-mm polyethylene insert, a 36 mm glenosphere, and a 29 mm glenoid baseplate	20° of anteversion, neutral version, 20° and 40° of retroversion	0°, 30°, and 60° of glenohumeral abduction in the scapular plane	Placing humeral component between 20° and 40° of retroversion restores a functional arc of motion without impingement
Kim, 2018 [30]	BS Study, BcS	Investigating impingement-free ROM of the gleno-humeral joint following RTSA with three types of implant models using computational motion analysis	7 patients	7 shoulders (retroversion)	GROUP I: AequalisTM-Reversed Shoulder System (Tornier Inc, Edina, MN, USA) GROUP II: AequalisTM-Reversed II Shoulder (Tornier, Amsterdam, The Netherlands) GROUP III: ComprehensiveTM Reverse Shoulder (Biomet Ortho-paedics, LLC, Warsaw, IN)	Humerus retroversion: -group I, 0°, 10°, 20° -group II, 0°, 10°, 20° -group III, 0°, 20°, 30°, 40°	For abduction, group III showed significantly greater mean maximal abduction compared to group II. No significant differences were found between Group I and II.	Humerus retroversion affected both IR and ER, especially in lateralization design (Group II and Group III).

Basic science (BS); biomechanical Study (BcS); case-series (CS); clinical-radiographical (CR); cohort (C); comparative (CO); level of evidence (LOE); prospective (PS); retrospective (RS); systematic review (SR). Prosthesis included. Delta III prosthesis (DePuy International, Leeds, UK)—Delta III RTSA (DePuy Orthopaedics, Inc., Warsaw, IN, USA)—Aequalis Reverse Shoulder (Tornier, Edina, MN, USA)—Comprehensive Reverse Total Shoulder Arthroplasty (Biomet, Inc., Warsaw, IN, USA)—Aequalis Ascend Reverse Flex convertible shoulder system—AequalisTM-Reversed II Shoulder (Tornier, Amsterdam, The Netherlands).

**Table 2:** Article conclusions on each topic.

Topic	Author, Year	Conclusion
**Impingement and range of movement**	Berton et al., 2015 [20]	Anteverted fixation maximized moment arm, but also increased inferior impingement. Moreover, 40° humeral retroversion displayed the largest ROM, while the 20° anteversion showed the smallest one
	Gulotta el al., 2011 [17]	Placing the implant in 0° to 20° of retroversion maximizes IR with the arm at the side. Concomitant latissimus dorsi transfer is recommended for patients with a deficient teres minor
	Henninger et al., 2012 [25]	RTSA reduced ER compared with intact shoulder. Deltoid tension and humeral version had little effect on ER
	Kim et al., 2018 [30]	Increasing humeral retroversion increases ER and decreases IR
	Kontaxis et al., 2017 [19]	Maximizing ROM by increasing humeral retroversion may not reduce the risk of impingement. An average 0° of humeral version should be preferred
	Lanzone et al., 2017 [28]	Restoring glenoid version around 0° yields the most optimal outcomes
	Permeswaran et al., 2017 [29]	Retroversion of the glenoid component reduces the risk of subluxation and increases ER and ROM
	Stephenson et al., 2011 [26]	Placing humeral component between 20° and 40° of retroversion restores a functional arc of motion without impingement
**Muscle forces**	Berton et al., 2015 [20]	The 0° and 20° retroversion was the optimum compromise between sufficient teres minor length and moment arm with minimum impingement
	Gulotta et al., 2011 [17]	Increasing retroversion did not affect the muscle force
	Henninger et al., 2012 [25]	Humeral version and implant thickness did not influence abduction forces
**Stability**	Favre et al., 2010 [18]	Effect of humeral component version was highly significant. Implanting the humeral component in neutral or some anteversion can improve anterior stability
	Henninger et al., 2012 [25]	Version and deltoid tension did not significantly alter dislocation forces

Basic science (BS); biomechanical study (BcS); case-series (CS); clinical-radiographical (CR); cohort (C); comparative (CO); level of evidence (LOE); prospective (PS); retrospective (RS); systematic review (SR).

## Data Availability

The data presented in this study are available on request from the corresponding author.
